# Assistance Dogs: Historic Patterns and Roles of Dogs Placed by ADI or IGDF Accredited Facilities and by Non-Accredited U.S. Facilities

**DOI:** 10.3389/fvets.2017.00001

**Published:** 2017-01-19

**Authors:** Sandra Walther, Mariko Yamamoto, Abigail Paige Thigpen, Anaissa Garcia, Neil H. Willits, Lynette A. Hart

**Affiliations:** ^1^Department of Population Health and Reproduction, School of Veterinary Medicine, University of California, Davis, Davis, CA, USA; ^2^Teikyo University of Science, Yamanashi, Japan; ^3^Department of Statistics, University of California, Davis, Davis, CA, USA

**Keywords:** Assistance Dogs International, autism dogs, diabetes dogs, hearing dogs, International Guide Dog Federation, mobility dogs, seizure dogs, service dogs

## Abstract

Dogs’ roles to support people with disabilities are increasing. Existing U.S. laws and regulations pertaining to the use of dogs for people with disabilities are only minimally enforced. Pushback legislation against some aspects of uses of assistance dogs currently is being passed or proposed in several states. Further, the U.S. Department of the Army and the Veterans’ Administration support only dogs trained by an Assistance Dogs International (ADI) or International Guide Dog Federation (IGDF) accredited facility. Lacking a mandatory national process for screening the selection, training, and placement of assistance dogs with persons who have disabilities, the U.S. offers a creative but confusing opportunity for people to train their own dogs for any disability. While no U.S. surveillance system monitors assistance dogs, other countries generally have a legislated or regulatory process for approving assistance dogs or a cultural convention for obtaining dogs from accredited facilities. We conducted an online survey investigating current demographics of assistance dogs placed in 2013 and 2014 with persons who have disabilities, by facilities worldwide that are associated with ADI or IGDF and by some non-accredited U.S. facilities. Placement data from ADI and IGDF facilities revealed that in most countries aside from the U.S., guide dogs were by far the main type of assistance dog placed. In the U.S., there were about equal numbers of mobility and guide dogs placed, including many placed by large older facilities, along with smaller numbers of other types of assistance dogs. In non-accredited U.S. facilities, psychiatric dogs accounted for most placements. Dogs for families with an autistic child were increasing in all regions around the world. Of dog breeds placed, accredited facilities usually mentioned Labrador Retrievers and Golden Retrievers, and sometimes, German Shepherd Dogs. The facilities bred their dogs in-house, or acquired them from certain breeders. Non-accredited facilities more often used dogs from shelters or assisted people in training their own dogs. Facilities in Europe and the U.S. place dogs in all roles; other parts of the world primarily focus on guide dogs. Expansion of assistance dogs in many roles is continuing, with numbers of dogs placed accelerating internationally.

## Introduction

The longstanding guiding role of a dog for a person with a visual impairment is obvious, and other roles of dogs have become evident. Guide dog owners report significantly increased social contacts and enhanced mental and physical well-being, as compared with visually impaired individuals without guide dogs. The close partnership is based on cooperative interactions between the person and dog in which they alternate the role of initiator for their joint actions (1). At the same time, guide dog owners walk faster and more efficiently than long-cane users (2). The very personal identity of the blind person changes to a person–dog team, with a softening of their former feelings of stigma (3). Reports describe the experience of owning a guide dog as life-changing, with both positive and negative consequences (4, 5). The loss of a guide dog at the end of a working partnership is especially difficult and distressing for the human partner (6).

Dogs to assist people with physical disabilities are referred to as mobility dogs. Canine Companions for Independence (CCI) (7) has placed almost 5,000 dogs since 1975, most for assistance with mobility. CCI mobility dog users report an increased sense of safety and peace of mind and greater independence. A systematic study examining the effects of service dogs for people with mobility (ambulatory) disabilities reviewed papers from the U.S., UK, and Japan and acknowledged the assistance with mobility while also highlighting the dogs’ roles for social participation and some psychological issues (8).

The value of guide dogs and mobility dogs goes beyond their performance of specific working tasks. They also promote self-confidence, peace of mind, greater independence, a sense of safety, and enhanced social interaction. This broader range of values has opened the door to other creative uses of dogs for people with special needs. These uses range from calming or protecting individuals with autism spectrum disorder or with post-traumatic stress disorder, to alerting people with diabetes to blood hypoglycemia and alerting people to a forthcoming seizure (9). The detection roles presumably stem from the dogs’ exceptional olfactory abilities and experimentally include detection of some types of cancer.

While taking a guide dog or mobility dog into a public place, airplane or train to help the disabled owner has generally been accepted because of the obvious need for the dog, there can be issues with regard to taking other types of assistance dogs onto planes, trains or into restaurants. To address these issues where the owner lacks a visibly apparent disability, most developed countries now have a centralized process by which persons with a disability can address specific requirements, often registering the disability and then legally registering the officially trained assistance dog that can be taken into public places and on airplanes, e.g., in Scotland (10). The international organizations, Assistance Dogs International (ADI) (11) and International Guide Dog Federation (IGDF) (12), have established basic guidelines for training, team training, and non-profit status to provide well-trained dogs to people with disabilities at low cost; each offers an accreditation process and posts the names of member facilities. Even with careful selection and training of dogs, predicting dogs that will be successful is challenging (13). As an alternative to legally requiring a registration process, countries may simply have a cultural convention for the person to partner only with a dog trained by an accredited facility.

The U.S. lacks a centralized process by which persons with a disability can legally register an assistance dog and take the dog into public places and on airplanes. Inconsistent nomenclature is used for the dogs’ various special roles (14), and the U.S. regulations on public access are complicated (15). U.S. Department of Justice (U.S. DOJ) regulations allow a person with a disability who has an assistance dog the privilege to take their dog into restaurants, planes, etc., requiring only that the person has a disability (physical, mental, or medical) and that the specially trained dog performs a task related to the disability (16, 17). Lacking any centralized legal system of registration, and no convention of only partnering with dogs from accredited facilities, the U.S. is a dynamic site for experimenting with, and developing, new roles for assistance dogs. Not being addressed here is further complexity that, in the U.S., people with emotional disabilities are allowed public access by the U.S. Department of Housing and Urban Development (HUD) in housing (18, 19) and by the U.S. Department of Transportation (DOT) on airlines (20) for their emotional support animals that do not have a specific, trained task. These rather liberal U.S. regulations regarding emotional support animals allow housing and airplane access for birds, monkeys, and even miniature horses that need not perform any tasks (21). The U.S. situation is confusing for persons having disabilities who seek to acquire a well-trained dog that can offer effective assistance.

Assistance dog placements and roles are growing rapidly in the U.S., where the focus is on providing equal or reasonable accommodation for people with disabilities. Regulations of the various U.S. agencies assure privacy for persons with disabilities and allow them to have full public access with dogs that are presumably trained for assistance tasks for persons having any of a broadly defined range of disabilities. The open-ended U.S. regulatory process for assistance dogs has allowed for creative development of new roles for assistance by dogs.

Assistance Dogs International specifies three main categories of assistance dogs: guide dogs for the blind, hearing dogs for the hard of hearing, and service dogs. Service dogs include varied roles, such as wheelchair assistance for mobility, epilepsy monitoring of seizures, aid for families with autistic children, hypoglycemic detection for diabetes, and psychiatric support. Whereas the term assistance dogs in the international ADI world refers to all specially trained dogs assisting persons with disabilities, one comparable term adopted in the U.S. is service dogs (16, 17), a term including all the roles just mentioned. However, one complication is that HUD recently began using the term assistance animal to also include an animal that provides emotional support to a person with a disability (21).

Department of Transportation (20) and HUD (18, 19) have sometimes suggested requiring increased scrutiny for the use of psychiatric service dogs in public places or housing. The Department of Veterans Affairs and the Department of the Army only provide financial support for personnel when the dogs were trained by ADI or IGDF accredited facilities: psychiatric dogs are excluded.

The names of ADI and IGDF facilities are listed online, but little information is readily available to the public on the numbers of dogs being placed for various assisting roles, or their breeds and sources. Guide Dog Users, Inc. (GDUI) (22) posted surveys from 13 U.S. facilities, including detailed information to inform prospective applicants. Those facilities ranged in number of placements from 6 to 310 dogs per year, with a median of 47 dogs. With the exception of a facility specializing for children, placements require applicants to be 16–18 years of age.

California offers optional free registrations of assistance dogs supplanting the need for licensing dogs, and some data are available for 1999–2012 registrations. By 2005, dogs of small body size were registered at a similar frequency as those of large body sizes (23). Chihuahuas became the most frequently registered small breed, and Pit Bulls (although not a recognized breed) became the fourth most frequently registered large “breed.” Demographics in psychiatric service dogs and emotional support service dogs changed rapidly; in early years of this study (2000–2002), psychiatric service dogs accounted for 17.1% of registrations and emotional support dogs were 0.0%, but in more recent years (2010–2012), psychiatric service dogs accounted for 31.9% of registrations and emotional support dogs were 19.0%.

In Europe, ADI lists 56 facilities in 19 countries; the Netherlands and Belgium each list 9 facilities, and the UK lists 8, whereas 8 countries list just one facility (11). The 51 facilities listed by IGDF are located in 20 countries; France has 12 facilities, and 10 countries have only one facility (12).

In Asia, ADI lists one facility in Japan and one in Taiwan that is also accredited with IGDF (11, 12). An additional facility for IGDF is in Korea, and one more in Taiwan, plus nine facilities in Japan. Thus, there is relatively little activity and research in Asia regarding assistance dogs apart from Japan. The legal qualification system under Japan’s Act on Assistance Dogs for Physically Disabled Persons specifies what is required for assistance dogs (24). When someone with a disability pairs with an assistance dog, the team needs to be assessed and qualified by one of a few designated organizations. To be qualified, the assisting behavior and health of the dog are assessed as well as the adequacy of the person with a disability in having the ability to accompany and handle the dog in public. Thus, not all people who are interested in living with an assistance dog can acquire one. While these governmental initiatives help assure order in public when assistance dogs are used in Japan, they also likely reduce the number of assistance dogs in Japan (25).

Additionally, Japanese historical, cultural, and environmental factors may also slow the development of assistance dogs in Japan (25). For example, training dogs for human use, including hunting and herding, was not historically common in Japan, and Japanese had relatively negative attitudes toward dogs having utilitarian roles compared to the UK (26, 27). In addition, the Japanese cultural characteristic regarding cleanliness requires that guide dog owners give a higher consideration for dogs’ excretions and shedding hair in public (28). The dense population and limited interpersonal space in homes in Japan amplify this concern. Little interest in acquiring a guide dog was also reported in Israel, where generally only veterans and members of upper strata used guide dogs (29). The authors attributed this to the great challenges in acquiring a dog and perhaps a cultural view by some of dogs as unclean.

Assistance Dogs International offers its certification process for non-profit facilities that place assistance dogs. Facilities initially enroll for candidate status and then work toward full accreditation. During this study’s data collection in 2015, the U.S./Canada region listed 98 accredited and candidate facilities and Europe listed 56. Guide dog facilities that are accredited with IGDF and that place dogs in additional roles beyond guiding also are eligible to seek ADI accreditation.

In this paper, we describe the breeds, sources, and numbers of dogs with each role placed in 2013 and 2014 worldwide by responding facilities of ADI or IGDF, and the year of each facility’s establishment. Additionally, we similarly surveyed responding non-accredited facilities in the U.S. that placed dogs.

## Materials and Methods

We first developed a brief survey with questions for each facility placing assistance dogs and uploaded it on SurveyMonkey^®^. Questions asked for contact and location information for the facility, the year of establishment, the total numbers of dogs placed in 2013 and 2014, and then the numbers of dogs placed each year for each category—guide dogs, hearing dogs, and service dogs for mobility, seizure alert, autism, psychiatric disorders, diabetic alert, and others. We asked whether handler-dog team training was provided, how long it lasts, and where it occurs. We asked the sources of the dogs and the breeds used. Finally, we asked the accreditation status of the facility, and for any comments. We contacted all accredited and candidate facilities worldwide that are associated with ADI and IGDF, sending them an email letter containing the survey link. Some facilities have dual accreditation; IGDF facilities placing guide dogs may apply for ADI accreditation if they place dogs in additional roles. This dual accreditation currently is held by eight North American facilities and five other international facilities. For non-responding ADI facilities in the U.S. and Europe, we accessed the facility’s year of establishment and identified a primary role of dogs that the facility placed, based on the information available on the facility’s website.

We also e-mailed a survey to all U.S. facilities that are not accredited that we could find listed online. To develop this list of 170 facilities, we searched by assistance dogs, service dogs, seizure dogs, diabetes alert dogs, autism dogs, PTSD dogs, and psychiatric dogs; we also gathered lists of facilities that were posted online. All of these facilities needed to be deleted if they also appeared on ADI or IGDF lists, or if they were duplicated.

We sent two reminder emails to all non-respondents and responded to numerous queries with follow-up replies and reminders. In addition, to gain maximal participation from ADI facilities in the U.S., we completed up to three phone calls to answer questions and remind non-responding U.S. facilities listed with ADI about the survey. Phone calls were not made to international facilities due to time zone and language differences.

We summarized the numerical information in several tables. ADI and IGDF present their members’ information by continent, and we followed that pattern for the introductory Table 1 and the final summarizing Figure 5, presenting North America, other international, and U.S. non-accredited facilities, to show the numbers of dogs by type placed around the world, and the extent to which current placements for each type are by older or newer facilities. Table 2 and the timelines also show the results by continent, but separate Canada and the U.S. This allows easy comparison between the accredited and non-accredited U.S. facilities and between the U.S. and Europe. Canada and the U.S. differ in their regulations. Further, focusing particularly on contrasts between Europe and the U.S. seems useful because other parts of the world have very few facilities, whereas the numbers of facilities are somewhat comparable between Europe and the U.S. Simple descriptive statistics describe uses, sources, and types of these assistance dogs being placed, and their patterns as related to geographic category, the facility’s age, and its accreditation status. To depict the numbers of dogs being placed in each role as related to the years the facilities were established, we created separate timelines for responding facilities in the various geographic areas (U.S., Canada, Europe, and other international countries) and non-accredited U.S. facilities. We plotted each facility on its geographic timeline by its year of establishment and indicated the roles and approximate total numbers of dogs placed, with the total numbers from 2013 and 2014 combined. To create separate timelines for non-responding accredited facilities in the U.S. and Europe, the regions having the greatest number of facilities, we used the date of establishment for each facility as indicated on their website and selected the most prominent role of dogs for the facility profiled online to show on the timeline.

**Table 1:** **Total numbers of dogs placed in 2013 and 2014, categorized by types of dogs, for accredited international or North American facilities, or non-accredited U.S. facilities**.

	Type of dog	# Dogs 2013/2014	% of Total dogs	# Facilities 2013/2014	Mdn dogs/year/facil (range of total dogs placed by all facilities/year)
International Assistance Dogs International and International Guide Dog Federation facilities (*n* = 34)	Guide	249/261	45	18/17	8.3 (1–77.5)
	Mobility	99/106	18	16/19	5.5 (1–14)
	Autism	53/67	10	9/11	4.3 (1.5–18)
	Hearing	118/141	23	3/5	9 (1–110)
	Psychiatric	3/20	2	2/6	1.5 (1–6.5)
	Diabetes	0/10	1	0/5	1 (0.5–2)
	Seizure	6/10	1	2/3	3.8 (1–5.5)
	Total	528/615 = 1,143	100	34	Mdn = 10 (1–110)

North American ADI and IGDF facilities (*n* = 55)	Guide	442/476	39	9/11	20 (2–199)
	Mobility	471/472	40	41/40	3.5 (1–177)
	Autism	95/110	9	18/19	3 (1–26.5)
	Hearing	59/50	5	8/7	4 (1–30)
	Psychiatric	52/67	5	14/16	2.3 (1–11)
	Diabetes	37/32	3	7/8	3 (1–10.5)
	Seizure	7/4	0	6/3	1 (1–2)
	Total	1,163/1,211 = 2,374	100	55	Mdn = 10 (1–233.5)

U.S. non-accredited facilities (*n* = 22)	Guide	2/1	0	1/1	1.5 (1.5–1.5)
	Mobility	59/52	14	15/12	2.5 (1–13)
	Autism	38/34	9	8/8	1.4 (1–14.5)
	Hearing	10/7	2	5/4	1.8 (1–3)
	Psychiatric	232/294	66	11/11	5 (1–136.5)
	Diabetes	17/23	5	6/4	3.5 (1–9.5)
	Seizure	15/13	4	6/5	2 (1–5)
	Total	373/424 = 797	100	22	Mdn = 8 (1–136.5)

**Table 2:** **Total Assistance Dogs International/International Guide Dog Federation dogs placed, by regions**.

Regions	Total dogs 2013/2014	Total dogs	# Facilities 2013/2014	Median dogs/facility in 2013/2014	% Increase 2013–2014
Europe	397/485	882	22/24	10/11	23
Australia/NZ	46/50	96	5/5	8/9	9
Asia	87/91	178	5/5	10/12	5
Canada	43/50	93	5/5	7/12	16
U.S.	1,120/1,153	2,273	49/48	5/6.5	3
Western	350/370	720	14/13	6/8	6
Central	315/310	625	11/11	5/6	−2
Southern	196/218	414	16/16	5/7	12
Eastern	259/255	514	8/8	22/17.5	−2

No personal information was obtained from any individual; only facilities were contacted and asked to provide information about the facility. Thus, IRB approval was not sought.

To assess the historical opening of facilities and the specific roles they primarily addressed in 2013 and 2014, the year of 1915 was taken as the recent, starting point year for the formal training of assisting dogs. Each facility was categorized by primary role of dogs it places and the years lapsed since 1915 until the facility was established; for Europe and the U.S., responding and non-responding ADI/IGDF facilities were combined, as they did not significantly differ. Kruskal–Wallis tests were employed to test for differences between Europe, the U.S./Canada, and other international facilities in establishment dates of facilities for each of the primary roles of dogs for which there were sufficient numbers (e.g., guide, mobility, and autism). None of these tests showed significant differences, so the specifics are not included in the results.

To assess the historical development of each of these dogs’ roles in various parts of the world (e.g., Europe, other international, and U.S./Canada), for each region, we listed all facilities placing a specific type of dog in 2013 in order of the facilities’ year of establishment. Then for each role, we determined the number of dogs placed by each facility during 2013 and computed the weighted median year of facility establishment, weighted by the median numbers of dogs placed. This measure was determined in each region—Europe, other international, and U.S./Canada—for the seven roles of dogs. It was calculated for responding ADI and IGDF facilities and then also for non-accredited U.S. facilities. Finally, a canonical correspondence analysis (CCA) was used to depict the relationships among data, especially the characteristics of accredited, candidate, and non-accredited facilities with respect to types of dogs they placed and sources of the dogs.

## Results

Among the 229 invitations sent to ADI or IGDF facilities, only one was returned due to an inactive email address with no forwarding suggestion. In contrast, among the 170 invitations sent to non-accredited facilities, 37 (22%) bounced back, suggesting a high rate of turnover. Response rates from ADI facilities were 35% internationally and 57% in North America; response rates from facilities only in IGDF were 16% and 25%, respectively. Considering only the invited non-accredited U.S. facilities whose invitations were not bounced back, the response rate was 17%.

### Characteristics of Facilities Internationally, in the U.S./Canada, and in Specific Geographic Regions

Accredited facilities in North America had a similar median number of dogs placed overall to those internationally, 10 per year (Table 1), whereas U.S. non-accredited facilities produced somewhat fewer dogs, 8 per year (Table 1). The ranges extended to 110 and 136.5 dogs placed per year for international and U.S. non-accredited facilities, respectively, whereas U.S. facilities extended to 233.5 dogs placed per year, reflecting some very large U.S. accredited facilities.

#### Accredited or Candidates: International (Excluding North America)

When considering only facilities outside North America, ADI and IGDF each list similar numbers of facilities, 68 (belonging to ADI and some also to IGDF) and 62 (belonging to IGDF only). Among the 34 responding facilities, close to 45% of the dogs placed in 2013 and 2014 were guide dogs (Table 1). Hearing dogs accounted for 23%. Mobility and autism dogs accounted for 18 and 10%, respectively; psychiatric, seizure, and diabetic alert each accounted for a small number of placements. The numbers of facilities involved in placing these types of dogs were similar for guide and mobility dogs, 18 and 19, respectively; the number of facilities placing autism dogs was next, and then hearing, psychiatric, diabetic and seizure dogs were each placed by only a few facilities. Generally, facilities placing hearing dogs placed the largest number of dogs in a specific role (Table 1: median = 9), guide dogs per facility per year were second, and facilities placing mobility dogs had the third largest number of placements. Autism and seizure placements per facility were next, and psychiatric and diabetic had a median of only 1–2 per facility. Overall, the international placements of dogs increased 16% in just the 1 year from 2013 to 2014.

#### Accredited or Candidates: U.S. and Canada

As shown in Table 1, and unlike the international facilities, the 55 responding North American facilities placed approximately equal numbers of guide and mobility dogs in 2013 and 2014, 39 and 40% of the total 2,374 dogs, respectively. Autism, hearing, psychiatric, diabetic alert, and seizure dogs accounted for the remaining 22% of the total, in that order. Mobility dogs were placed by the most facilities, by far, with autism and psychiatric dogs next. The guide dog facilities each placed a median of 20 guide dogs, several fold more than the facilities placed for other roles. The overall increase in number of North American placements from 2013 to 2014 was a modest 4%.

#### Non-Accredited: U.S.

As shown in Table 1, the 22 responding non-accredited U.S. facilities had a strong majority of placements of psychiatric dogs (66%). Remaining placements were for mobility, autism, diabetic alert, seizure, hearing, and guide dogs, in that order. Mobility dogs accounted for the greatest number of facilities, and psychiatric dogs were next, with autism, seizure, diabetic, hearing, and guide dogs following. The median number of dogs placed per facility for any particular role was only a few, with psychiatric being the highest (median = 5). The total number of dog placements increased 14% at these facilities from 2013 to 2014.

#### Geographic Regions

As shown in Table 2, among geographic regions, facilities in Europe reported the highest rate of growth from 2013 to 2014, with an overall 23% increase in total numbers of dogs placed. The five respondents each from Australia and Asia also increased their numbers of dogs placed, by 9 and 5%, respectively. Five respondents from Canada reported a 16% increase.

The number of responding ADI or IGDF facilities in the U.S. was more than double the number from Europe. Yet, overall, the U.S. had only a 3% increase in total number of dogs placed in 2014 as compared with 2013. In fact, the Central and Eastern states each reported a decline of 2% in their numbers of dogs placed. Facilities in the Eastern states differed from other regions in placing a large number of dogs per facility, reflecting their large guide dog facilities (medians 22 and 17.5 for 2013 and 2014). Southern states had the greatest increase in total number of dogs placed in 2014 compared with 2013, 12%, and the Western states were intermediate, 6%.

### Characteristics of Facilities by Their Year Established: Numbers and Roles of Dogs

Examining changes over time, we considered facilities that were established: prior to 1980, when primarily only guide dogs and hearing dogs were placed; 1981–2000, a period when the new service roles were developed; and 2001–2014, when the new service roles continued growing.

#### Facilities Established Prior to 1980

**Figure 1:**
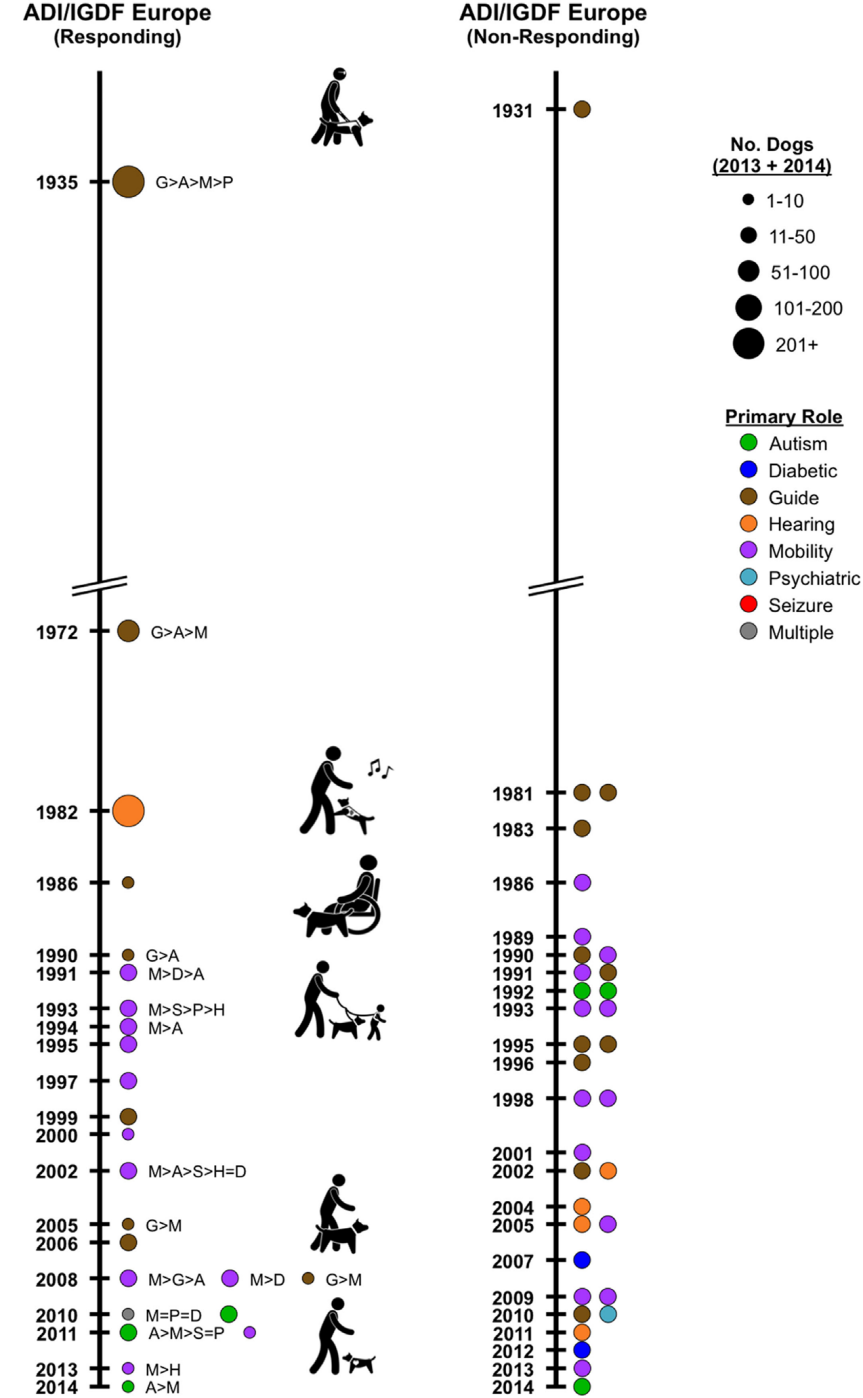
**Timeline depicting Assistance Dogs International or International Guide Dog Federation facilities in Europe, by year of establishment**. *Left*: responding facilities. Colored circles represent the primary type of assistance dogs placed; the circle size indicates the numerical range for the total dogs placed by the facility in 2013 and 2014. If more than one type of dog is placed, letters indicate the types placed, from most to least. *Right*: non-responding facilities. Year of establishment and a major type of dogs placed were acquired recently from facilities’ websites; no information is available on numbers of dogs placed.

**Figure 2:**
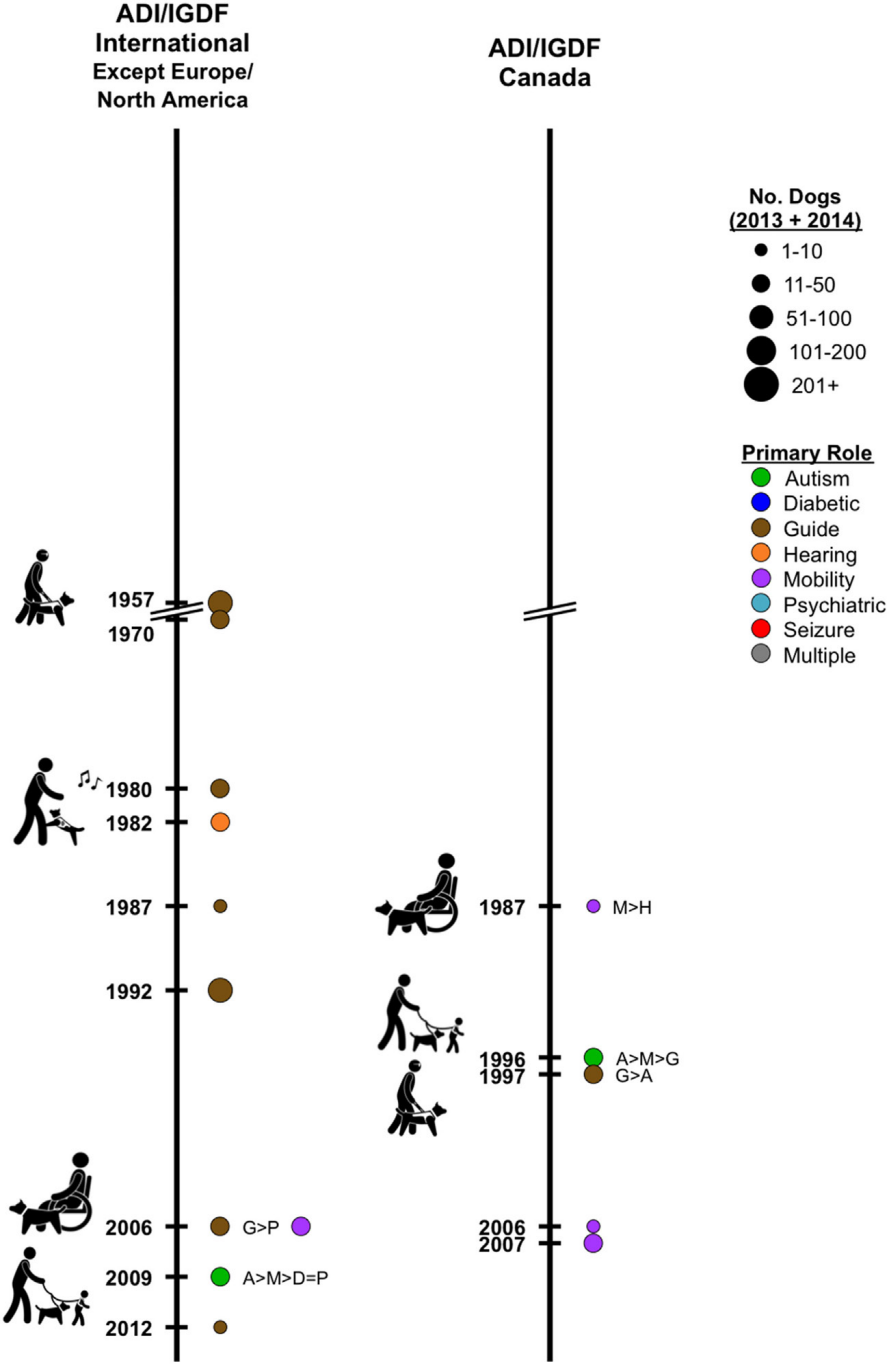
***Left*: timeline depicting responding International Assistance Dogs International or International Guide Dog Federation facilities, except in Europe or North America, by year of establishment**. Colored circles represent the primary type of assistance dogs placed; the circle size indicates the numerical range for the total dogs placed in 2013 and 2014. If more than one type of dog is placed, letters indicate the types placed, from most to least. *Right*: similar timeline for Canada.

Except for the U.S., the pioneering facilities that were established early generally continue to focus primarily on placing traditional guide dogs (Figure 1: Europe; Figure 2 left: international—Australia and Asia); but a few respondents in Europe now also place dogs for mobility assistance and for families with autistic children. These dogs often were bred in-house. No reporting Canadian facilities were yet established in this early period.

**Figure 3:**
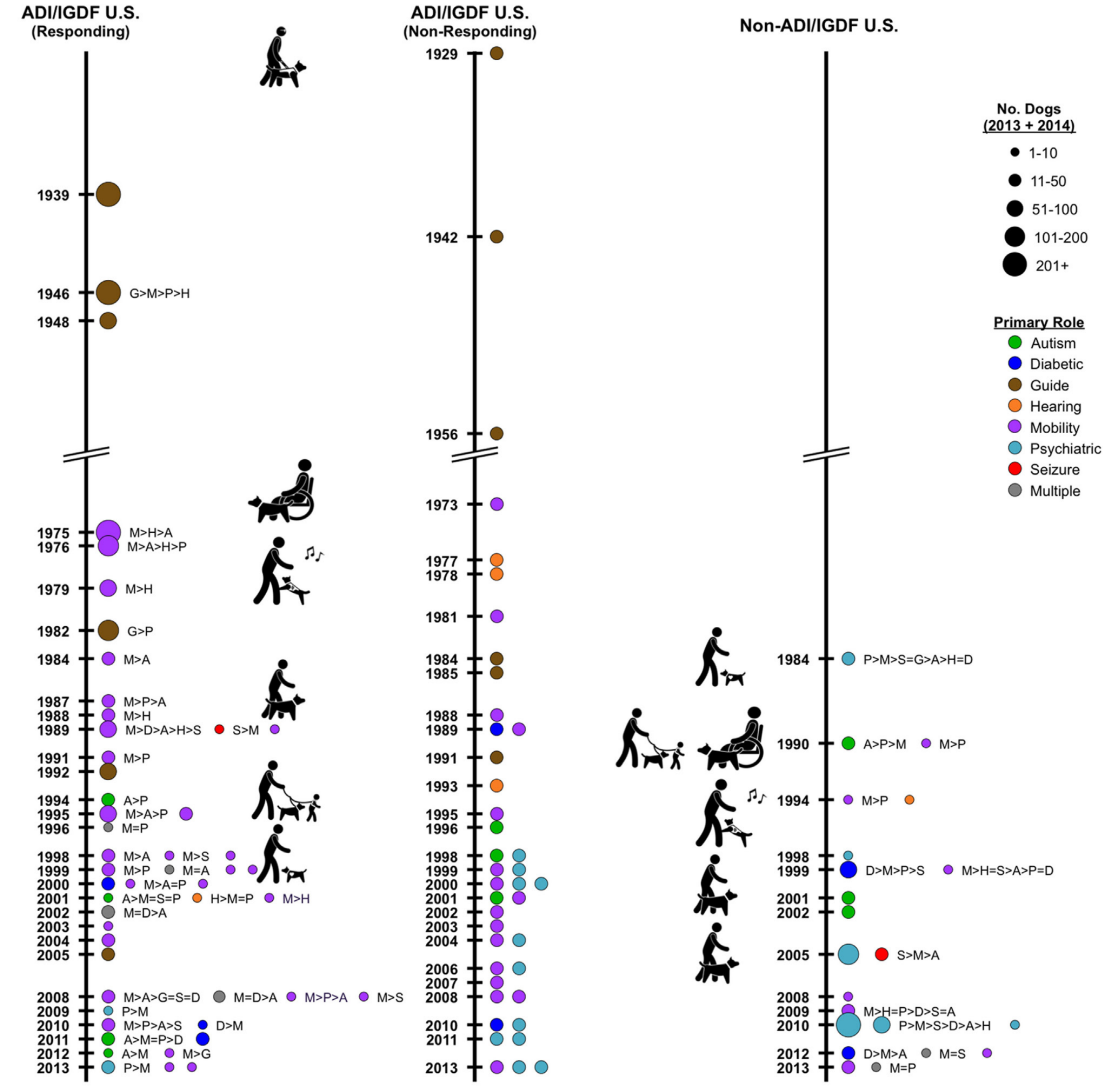
**Timeline depicting Assistance Dogs International or International Guide Dog Federation facilities in the U.S. by year of establishment**. *Left*: responding facilities. Colored circles represent the primary type of assistance dogs placed; the circle size indicates the numerical range for the total dogs placed in 2013 and 2014. If more than one type of dog is placed, letters indicate the types placed, from most to least. *Center*: non-responding facilities. Year of establishment and a major type of dogs placed recently were acquired from facilities’ websites; no information is available on numbers of dogs placed. *Right*: timeline depicting responding non-accredited facilities in the U.S. by year of establishment.

In the U.S., three responding accredited facilities, already established by 1948, continued placing primarily guide dogs, though one also diversified and placed mobility, psychiatric and hearing dogs (Figure 3 left). Three responding facilities established in the 1970s now place primarily mobility dogs, but all also placed hearing dogs, two placed autism dogs, and one placed psychiatric dogs. Similarly, three non-responding, accreditation facilities established by 1956 place guide dogs (Figure 3 center). Of three non-responding accreditation facilities established in the 1970s, one in 2016 profiles placing mobility dogs, and two profile hearing dogs. None of the responding, non-accredited U.S. facilities was established prior to 1980.

#### Facilities Established 1981–2000

Of the three international responding facilities in this category and outside Canada and Europe, two currently placed only guide dogs and one placed only hearing dogs (Figure 2 left). The 10 responding facilities in Europe placed hearing (1), primarily guide (3), and mobility (6) dogs (Figure 1 left). Autism (3) and psychiatric dogs (1) were also mentioned. The 18 non-responding facilities in Europe primarily profile in their websites, guide (8), mobility (8), psychiatric (1), and autism (1) dogs (Figure 1 right). Of the three responding accreditation facilities from Canada, mobility, autism, and guide dogs were each primary from one facility (Figure 2 right: Canada).

Twenty-three U.S. ADI facilities responded, including three guide dog facilities, two of which place a large number of dogs (Figure 3 left). Eighteen of these facilities placed mobility dogs, usually as a primary role for the facility. Other roles are autism (8), psychiatric (8), seizure (3), hearing (2), and diabetes (1). In the U.S., 17 non-responding accreditation facilities profile mobility (6), psychiatric (4), guide (3), autism (2), diabetes (1), and hearing (1) dogs (Figure 3 center). These facilities typically obtained their dogs from outside breeders, or in the case of dogs for guide work, dogs were bred in-house. Placements of hearing dogs were not increasing but were being continued, primarily by a small number of long-established facilities that seemed unlikely to add new roles for the types of dogs they placed.

Eight responding non-accredited U.S. facilities were established in this time frame (Figure 3 right). One facility placed solely hearing dogs. The others placed some psychiatric (7) dogs; facilities also placed mobility (6) and autism (3) dogs, as well as some diabetes, guide, and seizure dogs.

#### Facilities Established 2001–2014

Of the four responding facilities in this category and outside the U.S./Canada and Europe, two primarily placed guide dogs (Figure 2 left). Mobility and autism dogs were each primary for one facility. The 12 responding facilities in Europe primarily placed mobility (6), guide (3), and autism (3) dogs (Figure 1 left). The 15 non-responding facilities in Europe profile mobility (5), hearing (4), guide (2), diabetic (2), psychiatric (1), and autism (1) dogs (Figure 1 right). Two responding Canadian facilities placed mobility dogs (Figure 2 right).

Of 21 responding U.S. ADI facilities, 18 placed some mobility dogs; other roles addressed by facilities were autism (8), psychiatric (7), diabetes (6), seizure (4), guide (3), and hearing (2) (Figure 3 left). Eighteen non-responding U.S. ADI facilities profile on their websites mobility (9), psychiatric (7), diabetic (1), and autism (1) dogs (Figure 3 center).

From 14 responding non-accredited U.S. facilities, mobility (9), autism (6), and psychiatric (5) dogs, and seizure, hearing, and diabetes dogs also were represented (Figure 3 right).

### Characteristics of Facilities by Accreditation Status

Breeds that were almost invariably mentioned by accredited facilities include Golden Retrievers and Labrador Retrievers, sometimes with crosses, and often German Shepherd Dogs. Although a few facilities favored another specific breed CCAs addressing breeds by role or geography were unremarkable for other breeds.

#### Accredited

**Figure 4:**
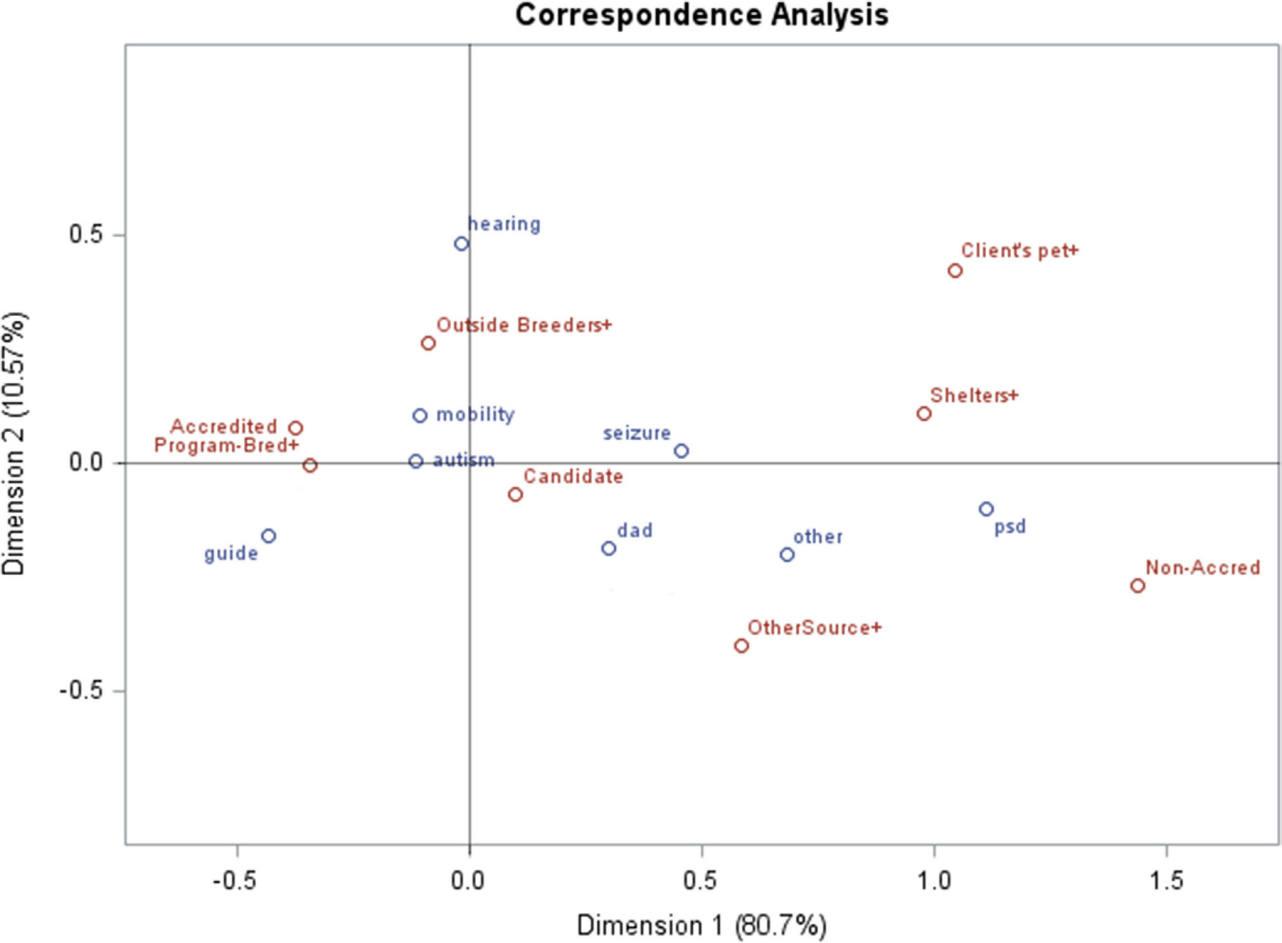
**Canonical correspondence analysis for responding Assistance Dogs International/International Guide Dog Federation facilities: for accreditation status, counts for types of dogs placed, and sources of dogs**.

As revealed in CCAs, fully accredited facilities very often bred their own dogs (Figure 4). Typically, they did not use dogs from shelters or assist persons in training their own dogs. They often placed guide, mobility, and hearing dogs.

#### Candidate

These facilities often placed diabetes, seizure, and autism dogs (Figure 4).

#### Non-Accredited

Facilities that are non-accredited often acquired dogs from shelters or worked with persons who trained their own companion dogs (Figure 4), as indicated by the distance of these variables from the origin, in a similar direction. Psychiatric dogs tended to be placed by non-accredited facilities, indicated by their colocation distant from the origin.

### Characteristics of Facilities throughout the World by the Development of Specific Roles of Dogs

**Figure 5:**
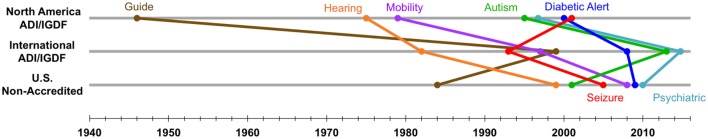
**Weighted median years of facility establishments (weighted by the median numbers of dogs placed in the seven roles during 2013), shown for accredited North American and other international facilities, as well as non-accredited U.S. facilities**.

The years of establishment for all facilities currently placing dogs in the various roles do not significantly differ for Europe and the U.S. However, the picture changes when considering the numbers of all dogs placed in the various roles and the years the relevant facilities were established. Figure 5 depicts the weighted median years of facility establishments (weighted by the median number of dogs placed in each of the seven roles at each facility during 2013), shown for accredited North American and International facilities, as well as non-accredited U.S. facilities. A few of North America’s facilities that were established early continue to have high outputs of assistance dogs, especially for guide dogs and mobility dogs, as compared with other international facilities, except for seizure dogs. On this measure, non-accredited U.S. facilities preceded the international facilities’ placements of numbers of guide, autistic, and psychiatric assistance dogs (Figure 5).

#### Guide Dogs

In the U.S., guide dogs often are trained and placed by large facilities (median per facility = 20 dogs/year) and the weighted median year of facility establishment for the median dog placed in 2013 was 1946. Internationally, facilities are smaller, placing a median number per facility of eight dogs per year, with the weighted median year of facility establishment placing the median dog being 1999. Even guide dogs are relatively new in some parts of the world, and several countries focus almost entirely on placement of only guide dogs. Responding non-accredited U.S. facilities placed only three guide dogs; although placing so few dogs, the weighted median facility year of establishment for the median dog placed was 1984.

#### Hearing Dogs

The weighted median year of facility establishment for placing the median hearing dog in 2013 in North America was 1975; the corresponding figure internationally is 1982. International respondents placed many more hearing dogs than those in North America (Table 1); Figure 1 reveals a large facility in the UK that places hearing dogs. Among the five responding, non-accredited U.S. facilities that placed hearing dogs, the weighted median establishment year of the facility placing the median dog was 1999.

#### Mobility Dogs

In North America, the weighted median year of facility establishment for placing the median mobility dog in 2013 was 1979, whereas, internationally, it was 1997. Among the 15 non-accredited U.S. facilities placing mobility dogs in 2013, the weighted median facility year placing the median dog was 2008.

#### Autism Dogs

The weighted median facility establishment year for placing the median autism dog in North America was 1995; internationally, it was 2013. Of eight non-accredited U.S. facilities placing autism dogs, the weighted median year was 2001.

#### Psychiatric Dogs

Similar to autism dogs, the weighted median facility establishment year for placing the median psychiatric dog in North America in 2013 was 1995; internationally, the corresponding year was 2013. Among 11 non-accredited U.S. facilities placing psychiatric dogs, the weighted median year for placing the median dog in 2013 was 2010.

#### Seizure Dogs

Among six responding facilities placing seizure dogs in 2013, the weighted median establishment year for placing the median dog in North America was 2001; the corresponding year internationally for the two facilities in 2013 was 1993. Six non-accredited U.S. facilities placed seizure dogs in 2013, and the weighted median facility year for placing the median dog was 2005.

#### Diabetes Dogs

Among seven responding facilities placing diabetes alert dogs in 2013, the weighted median year of facility establishment in North America placing the median dog was 2000. Internationally in 2013, no reporting facilities placed diabetes dogs, but by 2014 (figures used only in this case for comparable data), among five facilities placing a few dogs, the facility’s establishment weighted median year for placing the median dog was 2008. Among six non-accredited U.S. facilities placing diabetes dogs, the weighted median year was 2009.

In general, many of North America’s accredited facilities that today place high numbers of assistance dogs were established prior to 2000. Although facilities for various dogs’ roles were also established in Europe prior to 2000, much of their growth in the numbers of dogs placed has come from the recent creation of facilities. In North American and non-accredited U.S. facilities, dogs for autism are being placed by long-established facilities, just following guide, hearing, and mobility dogs, but dogs for autism placements began later at international facilities. Relative to other roles of dogs, North American facilities were somewhat delayed in placing dogs for seizure detection. Dogs for diabetes primarily have arisen since 2000. In countries beyond North America and Europe, placement of dogs has proceeded more slowly with a primary emphasis continuing on the role of dogs as guides.

## Discussion

The past decade or two in the U.S., Canada, and Europe have seen major increases in uses of assistance dogs for improving the function, health, and well-being of their human companions. Data-based studies document benefits not just for the people with visual impairments and those using wheel chairs but for seizure alerting, hypoglycemia detection, and comforting children with autism or adults with post-traumatic stress disorder (9, 15). In the discussion below, we address the worldwide regional differences in assistance dog roles, the differences and similarities in training facilities placing assistance dogs for the ever-expanding roles they have in society. With appropriate attention to the roles of assistance dogs in public places and transportation—protecting the comfort of the public—these canine companions can add immeasurably to the health and well-being of people in an increasing number of ways.

### Characteristics of Facilities Internationally, in the U.S., and in Specific Geographic Regions

Some European countries and the U.S. are increasingly welcoming to dogs in public areas. Asia is less accepting of dogs in public, which may affect the regional differences in the development of assistance dogs. Equal accommodation for people with disabilities developed in the U.S. alongside their growing expectations for individually trained dogs; the public access that was previously allowed pet dogs was insufficient for people who had assistance dogs supporting them. Therefore, DOJ (17) had to specifically differentiate the public access rights for handlers of service dogs versus pet dogs. However, in Europe, people were already allowed to accompany their pet dogs in various public locations. Thus, for some time Europeans did not need to create a special law for assistance dogs until the numbers and roles of assistance dogs expanded. In Asian countries, especially Japan, using dogs for assistance was uncommon; clear strict rules needed to be created to introduce assistance dogs into Japanese society and foster the understanding and tolerance of the public for dogs. This may explain why the expansion of assistance dogs developed early in the U.S., whereas recently, the growth has slowed and the U.S. shows a very low percentage increase from 2013 to 2014—only 3%. Some of the tapering off in the U.S. may be related to its growing number of non-accredited facilities. The highest rate of expansion—23%—is in Europe, whereas Asia remains slow in adopting varied assistance dogs, with a 5% increase from 2013 to 2014 (Table 2; Figure 2).

### Characteristics of Facilities by Accreditation Status

The organizational strength of accredited facilities accounts for some of their stability, accomplishments, and growth. Not only are they proficient in their ability and infrastructure for training dogs but also they maintain the financial power and human resources that are required to be accredited by ADI and IGDF, always preparing required documents and inviting inspectors from ADI or IGDF. This helps explain why the accredited facilities are placing many more dogs of most types than the non-accredited U.S. facilities. Although there are numerous non-accredited facilities, they inevitably suffer high turnover with financial and staffing struggles as reflected in the high level of bounced back email messages, and a few of these facilities place unqualified dogs. Non-accredited facilities have no obligation to be non-profits, so occasional unscrupulous persons can exploit unwary people seeking assistance dogs. Members of the public who are seeking dogs do not necessarily know about accreditation and may pay a large fee for a dog that proves not to be useful in assisting with a specific disability.

Although guide dog facilities are established in Asia, few people acquire guide dogs. Studies of obstacles to acquiring dogs in Japan show that people with visual disabilities feel that information resources pertaining to guide dogs are limited (30). Also, despite favorable legislation and due to low cultural acceptance of dogs in public, guide dog users in Japan describe stressful experiences when taking their dogs out on a rainy day, using a lavatory, or going on an outing (31). Placement of service dogs in Japan is proceeding very slowly despite evidence of positive functional and mental effects for people partnering with service dogs (32).

Working success of dogs is a challenge: half of the IGDF facilities surveyed to assess the working success of German Shepherd Dogs, Labrador Retrievers, Golden Retrievers, and Labrador × Golden Retriever crosses found diminished working success for the Labrador × Golden crosses (33). Using external breeders and assessing the dogs in field tests were associated with greater working success. These breeds are consistent with the accredited facilities in our study and similar to those reported by the 13 GDUI (22) facilities, although Dobermans, Poodles, and Bernese crosses were also mentioned there.

A limitation of this study was the low response rate from the non-accredited facilities in the U.S. We experienced the high turnover rate and frequent difficulty in reaching these facilities: presumably, the facilities that responded represented those with greater efficacy, stability, and resources.

Lacking professional centralized guidance for assistance dogs in the U.S., the widespread lack of knowledge people have about assistance dogs creates problems for everyone involved. Businesses and landlords often are unaware of the requirements to create access for handlers with their dogs, or which questions can be asked of someone with an assistance dog. People considering acquiring an assistance dog may simply get one to self-train without realizing that the dog will not provide meaningful assistance for their particular needs. A further burden faced by the growing number of persons training their own dogs and unaccredited U.S. facilities acquiring dogs from shelters to train is the poor predictive value of screening tests to select dogs. One small study found no correlation between a dog’s performance on the selection test and its ability to successfully complete a retrieval task for someone using a wheelchair (34).

### Characteristics of Facilities throughout the World by the Development of Specific Roles of Dogs

Numerous historic accredited facilities continue to place large numbers of guide or service dogs, and also often account for the increasing number of placements of dogs for families with an autistic child. Arguably this is a new role for dogs that is now in the mainstream for accredited facilities, going beyond the uses of dogs for diabetes, seizure, or psychiatric needs. As studies documented in Canada and Ireland, the dogs for autism ensure the safety of the child, while also enhancing the freedom and well-being of the family (35, 36). This use poses special challenges of welfare for the dogs; the dogs are likely to bond more with a parent than the child, and the child’s behavior and schedule may cause welfare burdens on the dog (37).

The placement of psychiatric dogs by accredited facilities has proceeded slowly while expanding more rapidly in the U.S. non-accredited facilities, where a majority of dogs placed assumed roles for psychiatric assistance. The distinction in the U.S. between psychiatric service dogs, emotional support dogs, and well-trained companion dogs for persons with mental illness can be confusing. Even ownership of pet dogs contributes toward the recovery from serious mental illness (38). Dogs in these roles serve as family and facilitate social connections with others, as has been well documented (39, 40).

A newer use of dogs is for medical alert, such as responding to low glycemia levels for persons with diabetes and under glucose control medication. Among 212 pet dog owners, 32% reported more than 10 incidents where the pet dog’s behavior changed in relation to hypoglycemia (41). With trained alert dogs, 8 of 10 responded appropriately to their owners’ blood glucose levels; they can assist with glucose control and increase the person’s independence (42). Alerting to impending migraine may be another alerting role for dogs that would provide time to preemptively treat migraines (43).

Guide dogs continue as the primary assisting role of dogs around the world. Assisting with mobility is a well-established role for dogs in North America that is increasing in Europe. Hearing dogs continue to be important in Europe but are somewhat eclipsed in the U.S. by new roles for dogs. Uses of dogs for families with an autistic child are steadily increasing throughout the world, and the placements often are by large accredited facilities.

Currently, with minimal U.S. enforcement of guidelines regarding the training and placement of assistance dogs and their access to public areas, restaurants, and airplanes, assistance dog facilities have already had a period of rapid growth. Studies show that assistance dogs play an essential role in human health and welfare. Further worldwide exploration of acceptable ways to integrate such dogs and other animals into the human health realm is still another angle on the “One Health” approach to medicine.

## Ethics Statement

The study included only census information on the numbers of dogs placed by assistance dog facilities. The study involved no direct involvement with the dogs or handlers, and thus no ethical review was required. We simply contacted the assistance dog facilities to acquire information on the dogs they have recently placed.

## Author Contributions

LH conceived the idea, oversaw data collection, and drafted manuscript. SW conducted all electronic communications with survey participants and prepared some final figures. AT prepared some final figures and edited all drafts. NW provided statistical guidance. MY participated in initial concept and survey design, assisted in initial manuscript draft, and reviewed all drafts. AG provided repeated contacts to facilities to remind them to participate and summarized data by regions.

## Conflict of Interest Statement

The authors declare that the research was conducted in the absence of any commercial or financial relationships that could be construed as a potential conflict of interest. The reviewer GW-S declared a shared affiliation, with several of the authors and a past collaboration with one of the authors (LH) to the handling Editor, who ensured that the process nevertheless met the standards of a fair and objective review.

## References

[1] Naderi SZ Miklosi A Koda A Csanyi V Co-operative interactions between blind persons and their dogs. Appl Anim Behav Sci (2001) 74:59–80. 10.1016/S0168-1591(01)00152-6

[2] Clarke-Carter DD Heyes AD Howarth CI The efficiency and walking speed of visually impaired people. Ergonomics (1986) 29(6):779–89. 10.1080/00140138608968314 3743536

[3] Sanders CR The impact of guide dogs on the identity of people with visual impairments. Anthrozoös (2000) 13(3):131–9. 10.2752/089279300786999815

[4] Whitmarsh L The benefits of guide dog ownership. Vis Impair Res (2005) 1:27–42. 10.1080/13882350590956439

[5] Wiggett-Barnard C Steel H. The experience of owning a guide dog. Disabil Rehabil (2008) 30(14):1014–26. 10.1080/09638280701466517 18953747

[6] Nicholson J The end of a partnership: the reactions of guide dog owners to the end of a working partnership with their guide dog. Br J Vis Impair (1993) 1:29–30.

[7] Canine Companions for Independence. (2015). Available from: http://www.cci.org/atf/cf/%7Bd369f549-15c4-46ee-bee3-52b190502f3f%7D/CCI%202015%20ANNUAL%20REPORT%20PDF%20FOR%20INTERACTIVE.PDF

[8] Winkle M Crowe TK Hendrix I. Service dogs and people with physical disabilities partnerships: a systematic review. Occup Ther Int (2012) 19:54–66. 10.1002/oti.323 21858889

[9] Hart LA Yamamoto M Dogs as helping partners and companions for humans. 2nd ed In: Serpell J, editor. The Domestic Dog. Cambridge, UK: Cambridge University Press (2016). p. 247–70.

[10] Refson K Jackson AJ Dusoir AE Archer DB The health and social status of guide dog owners and other visually impaired adults in Scotland. Vis Impair Res (1999) 1(2):95–109. 10.1076/vimr.1.2.95.4411

[11] Assistance Dogs International (ADI). (2016). Available from: http://www.assistancedogsinternational.org/members/programs-search/

[12] International Guide Dog Federation (IGDF). (2016). Available from: http://www.igdf.org.uk/links/

[13] Tomkins LM Thomson PC McGreevy PD Behavioral and physiological predictors of guide dog success. J Vet Behav (2011) 6:178–87. 10.1016/j.jveb.2010.12.002

[14] Parenti L Foreman A Meade BJ Wirth O. A revised taxonomy of assistance animals. J Rehabil Res Dev (2013) 50(6):745–56. 10.1682/JRRD.2012.11.0216 24203538 PMC4540185

[15] Hart L Yamamoto M Recruiting psychosocial health effects of animals for families and communities: transition to practice. 4th ed In: Fine A, editor. Handbook on Animal-Assisted Therapy: Theoretical Foundations and Guidelines for Practice. Amsterdam, Netherlands: Academic Press (2015). p. 53–72.

[16] U.S. Department of Justice (DOJ). Part 35. Nondiscrimination on the Basis of Disability in State and Local Government Services (as Amended by the Final Rule Published on September 15, 2010). (2010). Available from: http://www.ada.gov/regs2010/titleII_2010/titleII_2010_withbold.htm

[17] U.S. Department of Justice (DOJ). ADA 2010 Revised Requirements. Service Animals. (2011). Available from: http://www.ada.gov/service_animals_2010.pdf

[18] U.S. Department of Housing and Urban Development (HUD). Joint Statement of the Department of Housing and Urban Development and the Department of Justice. Reasonable Accommodations under the Fair Housing Act. (2004). Available from: https://www.hud.gov/offices/fheo/library/huddojstatement.pdf

[19] U.S. Department of Housing and Urban Development (HUD). 24 CFR Part 5. Pet Ownership for the Elderly and Persons with Disabilities; Final Rule. (2008). Available from: https://www.hud.gov/offices/fheo/FINALRULE/Pet_Ownership_Final_Rule.pdf

[20] U.S. Department of Transportation (DOT). Disability Issues: DOT Rule (Part 382). (2008). Available from: http://airconsumer.ost.dot.gov/ACAAcomplaint.htm

[21] U.S. Department of Housing and Urban Development (HUD). Service Animals and Assistance Animals for People with Disabilities and HUD-Funded Programs. (2013). Available from: https://www.ahead.org/uploads/conference/2013/handouts/2.10%20Service%20Animals%20Ackerman/HUD_ServiceAssistanceAnimals%20April%2025%202013.pdf

[22] Guide Dog Users, Inc. (GDUI). (2016). Available from: http://guidedogusersinc.org/resources/gdui-school-survey/

[23] Yamamoto M Lopez MT Hart LA Registrations of assistance dogs in California for identification tags: 1999-2012. PLoS One (2015) 10(8):e0132820 10.1371/journal.pone.0132820 26287610 PMC4544881

[24] Ministry of Health, Labour, and Welfare. Act on Assistance Dogs for Physically Disabled Persons. (In Japanese). (2011). Available from: http://law.e-gov.go.jp/htmldata/H14/H14HO049.html

[25] Yamamoto M Hart LA Ohta M Matsumoto K Ohtani N Obstacles and anticipated problems associated with acquiring assistance dogs, as expressed by Japanese people with physical disabilities. Hum Anim Interact Bull (2014) 2(1):59–79.

[26] Miura A Bradshaw JWS Tanida H Attitudes towards dogs: a study of university students in Japan and the UK. Anthrozoös (2000) 13(2):80–8. 10.2752/089279300786999860

[27] Miura A Bradshaw JWS Tanida H Attitudes towards assistance dogs in Japan and the UK: a comparison of college students studying animal care. Anthrozoös (2002) 15(3):227–42. 10.2752/089279302786992496

[28] Koda N Kubo M Ishigami T Furuhashi H Assessment of dog guides by users in Japan and suggestions for improvement. J Vis Impair Blind (2011) 105(10):591–600.

[29] Deshen S Deshen H On social aspects of the usage of guide-dogs and long-canes. Sociol Rev (1989) 37(1):89–103. 10.1111/j.1467-954X.1989.tb00022.x

[30] Yamamoto M Hart LA Matsumoto K Ohta M Ohtani N Japanese people with vision disabilities rate their experiences with information resources pertaining to guide dogs. Int J Orient Mobil (2013–2014) 6(1):70–82.

[31] Matsunaka K Koda N Acceptance of dog guides and daily stress levels of dog guide users and nonusers. J Vis Impair Blind (2008) 102(5):295–304.

[32] Shintani M Senda M Takayanagi T Katayama Y Furusawa K Okutani T The effect of service dogs on the improvement of health-related quality of life. Acta Med Okayama (2010) 64(2):109–13. 20424665 10.18926/AMO/32851

[33] Batt L Batt M Baguley J McGreevy P Relationships between puppy management practices and reported measures of success in guide dog training. J Vet Behav (2010) 5:240–6. 10.1016/j.jveb.2010.02.004

[34] Weiss E Greenberg G Service dog selection tests: effectiveness for dogs from animal shelters. Appl Anim Behav Sci (1997) 53:297–308. 10.1016/S0168-1591(96)01176-8

[35] Burrows KE Adams CL Spiers J Sentinels of safety: service dogs ensure safety and enhance freedom and well-being for families with autistic children. Qual Health Res (2008) 18:1642–9. 10.1177/1049732308327088 18955467

[36] Smyth C Slevin E Experiences of family life with an autism assistance dog. Learn Disabil Pract (2010) 13(4):12–7. 10.7748/ldp2010.05.13.4.12.c7758

[37] Burrows KE Adams CL Millman ST Factors affecting behavior and welfare of service dogs for children with autism spectrum disorder. J Appl Anim Welf Sci (2008) 11:42–62. 10.1080/10888700701555550 18444026

[38] Wisdom JP Saedi GA Green CA Another breed of “service” animals: STARS study findings about pet ownership and recover from serious mental illness. Am J Orthopsychiatry (2009) 79(3):430–6. 10.1037/a0016812 19839680 PMC2854030

[39] Hart L Hart B Bergin B Socializing effects of service dogs for people with disabilities. Anthrozoös (1987) 1(1):41–4. 10.2752/089279388787058696

[40] Lane DR McNicholas J Collis GM Dogs for the disabled; benefits to recipients and welfare of the dog. Appl Anim Behav Sci (1998) 59:49–60. 10.1016/S0168-1591(98)00120-8

[41] Wells DL Lawson SW Siriwardena AN. Canine responses to hypoglycemia in patients with type 1 diabetes. J Altern Complement Med (2008) 14:1235–41. 10.1089/acm.2008.0288 19040375

[42] Rooney NJ Morant S Guest C Investigation into the value of trained glycaemia alert dogs to clients with type 1 diabetes. PLoS One (2013) 8(8):e69921 10.1371/journal.pone.0069921 23950905 PMC3737201

[43] Marcus DA Canine responses to impending migraines. J Altern Complement Med (2012) 18(2):106–8. 10.1089/acm.2011.0773 22339098

